# Responses of soil bacterial communities to precipitation change in the semi-arid alpine grassland of Northern Tibet

**DOI:** 10.3389/fpls.2022.1036369

**Published:** 2022-10-17

**Authors:** Xueqin Li, Yan Yan, Xuyang Lu, Lijiao Fu, Yanling Liu

**Affiliations:** ^1^ Key Laboratory of Mountain Surface Processes and Ecological Regulation, Institute of Mountain Hazards and Environment, Chinese Academy of Sciences, Chengdu, China; ^2^ University of the Chinese Academy of Sciences, Chinese Academy of Sciences, Beijing, China

**Keywords:** Precipitation change, Alpine grassland, soil microbial, community structure, co-occurrence network

## Abstract

A change in precipitation can profoundly change the structure of soil microbial communities, especially in arid and semi-arid areas which are limited by moisture conditions. Therefore, it is crucial to explore how soil bacterial community composition and diversity will respond to variation in precipitation. Here we conducted a precipitation control experiment to simulate precipitation change by reducing and increasing rainfall by 25%, 50%, and 75% in the alpine grasslands of northern Tibet. The composition, diversity, and species interaction network of soil microbial community were studied by high-throughput sequencing, and the relationship between microbial community species and soil environmental factors was analyzed. Our results showed that Proteobacteria (45%–52%) and *Actinobacteria* (37%–45%) were the dominant bacteria in the soil. The alpha diversity index based on Shannon, Chao1, and Simpson indices revealed that precipitation change had no significant effect on richness and evenness of soil microbial communities. Non-metric multidimensional scaling (NMDS) and analysis of similarities (ANOSIM) showed that a clear separation of soil microbial communities between D2(-50%),D3(-75%) and W2(+50%), W3(+75%) treatments. The microbial interaction network indicated that the water-increasing treatment group had closer connections, and *Proteobacteria* and *Actinomycetes* were the core species. Furthermore, there was a stronger positive correlation between species in the water-reducing treatment group, the contribution of *Proteobacteria* decreased significantly, the role of connecting hub decreased, and *Actinomycetes* became the most important core microbial species. In addition, soil water content (SWC) and available phosphorus (AP) were closely related to the variations in soil microbial compositions. The findings of this study provide a theoretical basis for the driving mechanism of global climate change on soil microbial community and grassland ecosystem in alpine grassland.

## Introduction

Ongoing global climate change is expected to alter soil microbial communities through direct and indirect means (Brockett et al., 2012). The direct means will change microbial metabolism and substrate availability, while the indirect means will affect the interaction between soil microorganisms and plants (Brockett et al., 2012; Classen et al., 2015). In the second case, the soil microbial community changes, which may contribute to changes in terrestrial ecosystem processes, include the decomposition of soil organic matter, carbon sequestration, and nutrient cycling (Allison and Treseder, 2008; Schindlbacher et al., 2011). These processes reflect climate change-induced changes in plant growth, phenology, and species composition. Precipitation change is the main content and research focus of global climate change (Nyström et al., 2019; Ault, 2020). Variations in precipitation and soil moisture are of great importance to microorganisms, and even have a greater impact than other consequences of global climate change, such as rising temperature and CO_2_ concentration (Schimel and Schaeffer, 2012). Therefore, understanding how soil microbial communities change with the increase or decrease of precipitation is the key to further explore the ecological physiological and biogeochemical trajectories (Felsmann et al., 2015).

At present, the characteristics of soil microbial community responses to precipitation change have been largely reported (Clark et al., 2009; Landesman and Dighton, 2010; Nielsen and Ball, 2015; Gao et al., 2016; Zhou et al., 2018), but the experimental results are very different. A study of drought simulation experiments in two continents, North America and Australia, showed that bacteria within the phyla *Acidobacteria, Chloroflexi*, and *Nitrospirae* were more abundant in conditions of decreased precipitation. These groups are oligotrophic organisms that grow slowly and tend to form small colonies. This allows them to maintain high abundance even in dry environments and have a strong tolerance to water stress (Evans and Wallenstein, 2014; Ochoa-Hueso et al., 2018). In addition, meta-analysis on the effects of global precipitation changes on soil microbial communities showed that reduced precipitation shifts bacterial communities to Gram-positive bacteria (with strong, thick, interconnected peptidoglycan cell walls), so that they can resist dry conditions in some ways (Uhlírová et al., 2005; Zhou et al., 2018). With the increase of precipitation, the relative abundance of *Proteobacteria* and *Bacteroidetes* gradually increased. This may be due to the fact that *Proteobacteria* and *Bacteroidetes* are generally considered to be symbiotic trophic organisms, which follow trends consistent with changes in ambient precipitation (Fierer et al., 2009; Li et al., 2022a). At the same time, soil microbial communities may respond differently in different ecosystems. In alpine steppe, there was no change found in species composition in bacterial communities after reduced precipitation (Williams, 2007). Studies in temperate steppe confirmed that precipitation can have a significant effect on the relative abundance of soil microorganisms, although it did not have a significant effect on their diversity (Zhao et al., 2016; Wang et al., 2017). Studies in desert grassland emphasized that the response of soil bacterial community diversity to precipitation gradient was not apparent or delayed (Banu et al., 2004; Cruz-Martínez et al., 2009; Bell et al., 2014; Yang et al., 2021). In general, soil microbial communities show different characteristics in response to precipitation increase/decrease and ecosystem differences. At present, there is no general consensus and understanding of regularity on the response of soil microbial communities to precipitation conditions and specific habitats, which needs to be further supplemented. Those studies will also help us better understand the water-related life strategies of soil bacterial communities.

Generally speaking, soil microorganisms form complex interspecific networks, which greatly regulate ecological community structure and ecosystem function, rather than living in isolation (He et al., 2017). Microbial co-occurrence networks have been widely used to study microbial interactions in ecosystems and to identify microbial keystone groups (Barberán et al., 2012; Williams et al., 2014; De Menezes et al., 2017; Bingjian et al., 2020), which play an important role in shaping community structure and driving ecosystem function (Banerjee et al., 2018; Mikhailov et al., 2019). The more compact and closely connected the network is, the more deterministic processes determine the composition of the microbial community; conversely, the looser and more isolated the network is, the more random processes determine the composition of the microbial community (Jiang et al., 2018). In summary, co-occurrence network analysis is an important means to understand microbial community structure and interactions between microorganisms (Barberán et al., 2012). For example, network analysis can predict under-characterized phyla between members to find novel interactions, thus providing valuable information for co-culture of different species (Faust and Raes, 2012). *Actinobacteria* was found to be the keystone phylum for connecting other bacterial members (Zhou et al., 2011). *Sordariales* and *Hypocreales* played similar “connecting” roles in one study of fungal co-occurrence networks (Lu et al., 2013). However, studies examining and comparing changes in network structure in response to altered precipitation regimes are still lacking, and information on the effects of climate change and microbial feedbacks in soil is limited.

Although we pay a lot of attention to microbial communities, abiotic factors such as soil temperature (Feng and Simpson, 2009), soil moisture (Brockett et al., 2012), and soil pH often play a significant role in driving changes in microbial community structure (Fierer and Jackson, 2006). In addition, affected by differences in soil water conditions, the dissolution of soil nutrients would be accelerated or slowed down, resulting in changes in soil nutrients, thus changing the structure of soil microorganisms (Parton et al., 1995; Zhang et al., 2005). Niu et al. (2021) found that nitrogen content was significantly correlated with *Fibrobacteres, Bacteroidetes, Patescibacteria, Gemmatimonadetes*, and *Actinobacteria*, while soil phosphorus content was also significantly correlated with *Fibrobacteres, Bacteroidetes*, and *Actinobacteria*. Soil available potassium was significantly related to the *Entomophthoromycota* and *Chytridiomycota*. Furthermore, soil available nitrogen was significantly associated with the phylum, *Entomophthoromycota*, while soil available phosphorus had a negative correlation with *Cryptomycota* (Niu et al., 2021)

The Qinghai-Tibet Plateau has become the region with the greatest uncertainty of environmental change due to global climate change (Tandong, 2019). The basic feature of climate change is warming and humidification. Recently, precipitation has shown an overall increase trend, with an increase of 2.2% every 10 years. Near- (present–2050) and long-term (2051–2100) precipitation is projected to increase by 10.4%–11% and 14.2%–21.4%, respectively, over the current base period (Chen et al., 2015). In the context of continuous global warming, precipitation has rapidly increased along with the accelerated warming of the Qinghai-Tibet Plateau, accompanied by the increase of extreme rainfall events (drought and rewetting), which will directly affect the plant species diversity and community composition of the plateau ecosystem (Zhang et al., 2020). However, due to the complexity of grassland ecosystems, how precipitation changes microbial communities and co-occurrence network patterns in alpine grassland is still controversial, which needs further exploration to deepen our understanding. In this study, we conducted a precipitation control experiment in the alpine steppe of northern Tibet to answer the following two questions:(1) how do the increase and decrease of precipitation change soil microbial community structure and co-occurrence network? (2) What factors drive the change of microbial community under precipitation change?

## Material and methods

### Study site

**Figure 1:**
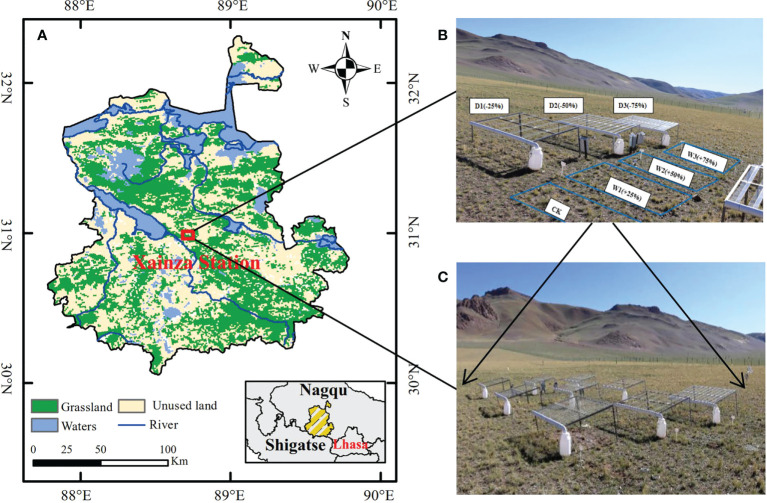
Location of the study area and design of the experiment. **(A)** Location of Xainza Station; **(B)** Diagram of the seven precipitation treatments; **(C)** Complete experimental site with three blocks and 21 plots.

The study was conducted in Xainza Alpine Grassland and the Wetland Ecosystem Observation and Experimental Station (30°57’N, 88°42’E, 4675 m above sea level) in Xainza County, Nagqu City, Tibet Autonomous Region (**Figure 1A**). The research area belongs to the semi-arid monsoon climate of the sub-frigid plateau with cold and dry climate. The annual average temperature is 0.4 °C, and the highest monthly average temperature is not higher than 14 °C (July) and the lowest is below 10 °C (January) (Xiong et al., 2016). The annual precipitation of 298.6 mm is concentrated in June to August. In addition, the annual evaporation is as high as 2181.1 mm, and the annual evaporation of rain is much greater than the annual precipitation (Ping et al., 2021). The vegetation type of the study area belongs to alpine steppe, and *Stipa Purpurea* community is the dominant community in this area. Associated plants include *Carex moocroftii*, *Artemisia Capillaris*, *Leontopodium nanum*, *Oxytropis microphylla*, *Kentucky bulegrass*, and *Stellera chamaejasme* (Ma et al., 2016b). Plants turn green in the middle and late may of each year, the growing period is from June to August, and gradually stops growing in September. In October, plants basically stop growing and enter the yellow stage, which lasts until the beginning of May of the following year. The main soil type is alpine steppe soil, with thin soil layer and easy erosion, and the soil has obvious coarse-grained characteristics (Cai et al., 2014).

### Experimental design

#### Precipitation gradient experiment

The precipitation control experiment was conducted with canopy (Kundel et al., 2018). Seven precipitation gradients were set according to the precipitation variability in the study area in the past 30 years. On the basis of natural precipitation (CK) increase 25%, 50% and 75% water (W1, W2, W3, respectively), decrease 25%, 50%, and 75% water (D1, D2, D3, respectively). We set up a total of seven precipitation gradients above to simulate different water conditions, and laid out three replicates for each gradient, a total of 21 quadrats, the size of each quadrat was 3 m×2 m. In order to avoid the marginal effect between plants and water infiltration between quadrats, the interval between two adjacent quadrats was set as 2 m. In addition, random block design was used for the layout of quadrats, and precipitation gradient experiment was carried out during the 2020–2021 growing season (From May to September) (**Figures 1B, C**).

**Water-reducing treatment:** Square steel welded at 8 cm × 8 cm was used as a rain shield. The two ends of the canopy were 0.7 m and 1.05 m from the ground respectively, so that the canopy was inclined. The transparent PVC drainage pipe (diameter of 12 cm) was cut along the diameter direction, so that it could become a rainwater collecting trough, and the top of each canopy was evenly fixed with 5 (D1), 10 (D2), 15 (D3) V-type acrylic plates to be interception of precipitation by 25%, 50%, and 75% of occlude sample area (Zhang et al., 2018b; Wang et al., 2020).

**Water-increasing treatment:** The rainwater collecting trough led the trapped rainwater through the water pipe to the rainwater collecting barrel. Then, 24 hours after the rainfall stopped, the 25%, 50%, and 75% of the collected precipitation in the water reduction area were uniformly introduced to the corresponding plots through drip irrigation pipes, so that the plots increased precipitation by 25% (W1), 50% (W2), and 75% (W3) to ensure the synchronicity of rainfall under each rainfall treatment.

#### Sample collection

In August 2021, we carefully excavated the rhizosphere soil of the dominant species (*Stipa purpurea*) in the community under seven precipitation gradients, with four soil samples from each quadrat, and a total of 28 soil samples were collected. We labeled the soil samples and placed them in a freezer to keep them cold until they were processed for metagenomic sequencing. At the same time, we also collected dry soil samples under seven precipitation gradients to determine soil physical and chemical properties. The roots and other debris in the soil were picked out, dried, ground, and screened through 200 mesh. The screened soil samples were taken to determine the physical and chemical properties of the soil, including pH, soil organic matter (SOM), total nitrogen (TN), total potassium (K), total phosphorus (P), available potassium (AK), available phosphorus (AP), and available nitrogen (AN). **Table 1** shows the method used to gather this data.

**Table 1:** Soil physical and chemical properties and determination methods.

Project	Method	Sources
pH	Extraction with distilled water removed from carbon dioxide	(Wang et al., 2014a)
SOM	Potassium dichromate external heating method	(Huang et al., 2020)
TN	Kjeldahl determination	(Liu, 2020)
K	Boiling nitric acid method	(Zhu et al., 2018)
P	Alkali fusion-molybdenum-antimony anti spectrophotometry	(Chen et al., 2018)
AK	Flame photometer	(Guo et al., 2012)
AP	Sodium bicarbonate extraction - molybdenum antimony resistance method	(Bu and Magdoff, 2003)
AN	Alkali hydrolysis diffusion method	(Ye and Zhao, 2011)

### DNA extraction and DNBSEQ sequencing of soil samples

The metagenomic sequencing of our soil samples was commissioned by BGI Shenzhen Co., Ltd. The experimental process was mainly divided into DNA extraction and DNBSEQ sequencing library construction. A 96-well deep-well plate was prepared and swabs were loaded from the soil sample. DNA was extracted from each soil sample using the magnetic bead method, and then purified automatically using the Kingfisher purification system. At the end of the above procedure, the DNA solution in the deep well plate was transferred to a 1.5-mL centrifuge tube for storage, in order to prepare the DNA solution for library construction and sequencing on DNBSEQ sequencing platform. Library construction of DNBSEQ sequencing platform mainly consists of nine steps, including sample testing, sample interruption, fragment size selection, end repair, preparation reaction system, PCR reaction and product recovery, product cyclization, library detection, and generation of sequencing data. The detailed process of each step has been previously described (Fu et al., 2022).

### Processing of sequencing data

To obtain clean data, the raw sequencing data were processed using SOAPnuke software as follows: (1) exclude reads containing 10% uncertain bases (N bases); (2) exclude reads containing adapter sequences (15 bases or longer sequence aligned to the adapter sequence); (3) exclude reads containing low-quality bases of 20% (bases of Q<20); (4) for the sample with the host or environment sources, a filtering step was added here to remove the host genome sequence to reduce interference of the host sequence on the subsequent analysis (Soap2, more than 90% similarity, a host reference sequence is required) (Li et al., 2009). High-quality short reads of each DNA sample were assembled by the MEGAHIT. The parameters of MEGAHIT was ‘megahit_opts = –min-count 2 –k-min 33 –k-max 53 –k-step 10 –no-mercy –memory 0.9 –min-contig-len 200 –num-cpu-threads 5’ (Li et al., 2015). MetaGeneMark was used for metagenomic genes prediction (Zhu et al., 2010). The assembled reads were clustered by CD-HIT to eliminate redundancy (Fu et al., 2012), and finally unigenes were obtained with a similarity threshold of 95%. The parameter of CD-HIT was ‘CD_HitOpts = -aS 0.9 -c 0.95 -M 0 -d 0 -g 1 -T 12’. Salmon software (version 1.4.0) was used to standardize sequences to determine gene abundance by the transcripts per million (TPM) method. Species annotations and species abundances were calculated using Kraken2(DB2020), and the filter options were ‘-l 20 -q 0.5 -n 0.1 -d -Q 2 -5 0’ (Wood and Salzberg, 2014).

Our raw data has been successfully uploaded to NCBI database, the accession number is PRJNA881773, and click on the link below to see “https://www.ncbi.nlm.nih.gov/sra/PRJNA881773”.

### Statistical analysis

One-way ANOVA was used to compare the relative abundance of phylum level and genus level species in different precipitation gradients. Three commonly used α-diversity indices of the bacterial community were calculated by QIIME (Version 1.80) (BGI Shenzhen Co., Ltd) (Caporaso et al., 2010). Chao1 is the species richness index, and its value largely reflects the variation of rare species (Chao and Bunge, 2002). Shannon index reflects species richness and evenness, and the individual weight of rare species is higher than that of common species (Hill et al., 2003). The Simpson index reflects the degree of consistency in a community (Zhong et al., 2010). The formula for calculating the α- diversity indices are as follows:


(1) $$S_{Chao1}=S_{obs}+\frac{F1\left(F1-1\right)}{2\left(F2+1\right)}$$

(2) $$H_{Shannon}=-\sum A_{i}^{*}\text{ln}(A_{i})$$

(3) $$D_{Simpson}=\frac{\sum_{i=1}^{S_{obs}}ni\left(ni-1\right)}{N\left(N-1\right)}$$

Where S_obs_ is the number of species observed in the sample; F1 and F2 represent the number of singletons and doubletons, respectively; N is the total number of individuals in all species; ni represents the total number of individuals of species i; Ai is the relative abundance of species.

β-diversity was represented by Bray–Curtis dissimilarity between different water conditions (Chen et al., 2019), and then pairwise differences between different water conditions in low-dimensional space were explained using non-metric multidimensional (NMDS). In addition, the analysis of similarity (ANOSIM) was used to determine whether the water gradients were significantly separated (Ren et al., 2018b). The graphs were generated using the “ggplot” package (Version 3.3.5). The microbial co-occurrence network was constructed based on data of species for the top 200 absolute abundance microbial genera after removing rare species (the number of detected 0 values more than 3) in the soil samples of seven precipitation gradients. A strong and significant correlation at the threshold of Spearman’s |r|>0.7 and p<0.01 were commonly used and recognized to be valid and robust (Steinhauser et al., 2008, Barberán et al., 2012, Ma et al., 2017). The nodes and edges file calculated using package “WGCNA” in R platform (http://www.r-project.org), and visualized in Gephi software (Version 0.9.3) (Bastian et al., 2009). The topological features of nodes-level (including clustering coefficient), edge-level (including path percentage of positive and negative edges, Average path length), and network-level (including network diameter, average degree, modularity) were calculated in Gephi (Bastian et al., 2009).

Redundancy analysis (RDA) was used to analyze the relationship between soil bacterial composition and environmental factors. The selected environmental factors included SWC, ST, pH, SOM, TN, AN, K, AK, P and AP. Monte Carlo permutation test was then used to test the significance of RDA results. The analysis process was performed in Canoco5.0. The map displaying geographical locations of the study area was constructed based on the remote sensing monitoring data of land use in Tibet in 2020 downloaded by the Resource and Environmental Science and Data Center and the software ArcGIS (version 10.5, Environmental Systems Research Institute, Inc., CA, USA).

## Results

### Soil bacterial community structures and diversity

#### Relative abundence

A total of 17,739,185,700–18,816,624,900 clean base with high-quality metagenome sequences were identified from the 28 samples from Northern Tibet. After chimera detection and removal, 503,015–1,040,699 contig numbers with an average size of 507–652 bp were obtained. Sequencing data of all soil samples showed that bacteria accounted for more than 98%, while archaea, eukaryotes, and viruses accounted for less than 2%. Therefore, the main research object of this paper was soil bacterial community.

**Figure 2:**
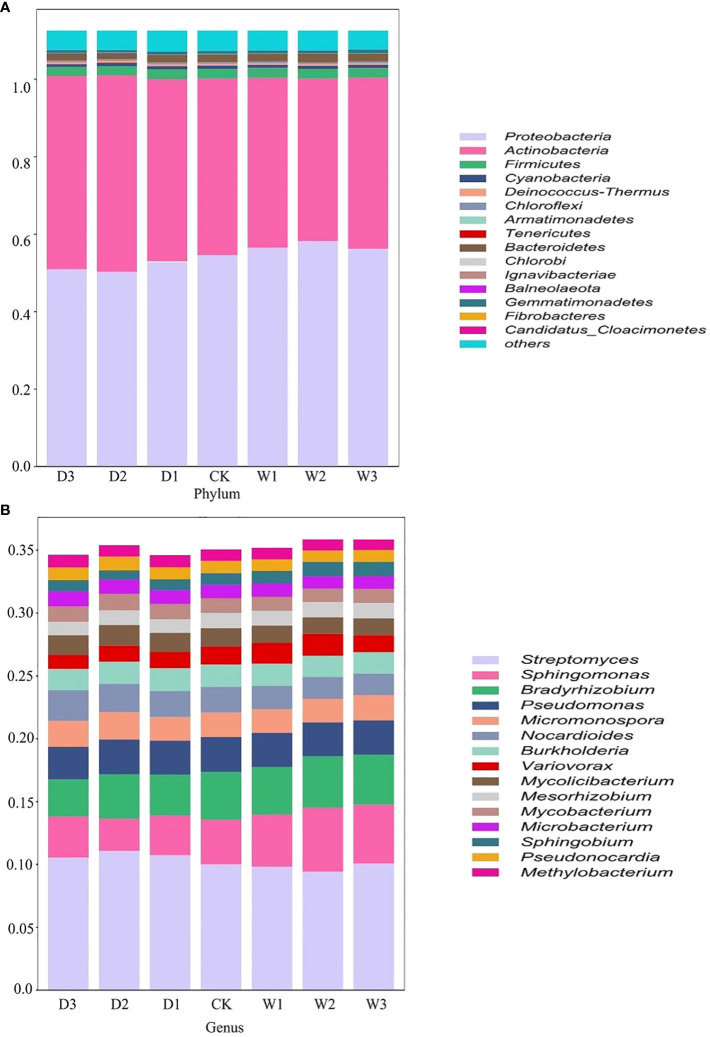
Relative abundance of bacteria in different precipitation gradients at the phylum and genus levels on the Northern Tibet. The bacteria whose relative abundance are in the top 15 at phylum **(A)** and genus **(B)** levels. D3, water reducing 75%; D3, water reducing 50%; D1, water reducing 25%; CK, control treatment; W1, water increasing 25%; W2, water increasing 50%; W3, water increasing 75%.

According to the relative abundance ranking of top15 taxa at phylum level (**Figure 2**), the bacteria with relative abundance ranking lower than 15 were classified as others, and the results showed that the bacterial proportion characteristics among the seven precipitation gradients were relatively consistent. *Proteobacteria* and *Actinobacteria* are the two most abundant phyla, accounting for 45%–52% and 37%–45% of all phylum, respectively. *Firmicutes* (2.1–2.3%), *Cyanobacteria* (0.64%-0.81%), and *Deinococcus-Thermus* (0.43%–0.47%) account for a very small proportion (**Figure 2A**). One-way ANOVA showed that *Proteobacteria, Actinobacteria*, and *Candidatus_Cloacimonetes* had significant differences among the seven precipitation gradients (**Table 2**). The relative abundance of *Proteobacteria* in water-increasing treatment was higher than that in water-reducing treatment, while the relative abundance of *Actinobacteria* was lower (P <0.05).

**Table 2:** Differences in community composition of phylum and genus of soil microorganisms under different water conditions.

Phylum	F	P	Genus	F	P
Proteobacteria	6.364	**0.001**	Streptomyces	2.814	**0.036**
Actinobacteria	4.516	**0.004**	Sphingomonas	13.124	**0.000**
Firmicutes	0.268	0.946	Bradyrhizobium	2.919	**0.031**
Cyanobacteria	0.459	0.831	Pseudomonas	0.896	0.516
Deinococcus-Thermus	1.668	0.178	Micromonospora	0.874	0.531
Chloroflexi	0.394	0.874	Nocardioides	7.442	**0.000**
Armatimonadetes	0.257	0.951	Burkholderia	1.495	0.228
Tenericutes	0.544	0.769	Variovorax	5.019	**0.002**
Bacteroidetes	1.745	0.160	Mycolicibacterium	10.624	**0.000**
Chlorobi	0.576	0.745	Mesorhizobium	0.939	0.489
Ignavibacteriae	2.266	0.076	Mycobacterium	7.299	**0.000**
Balneolaeota	1.101	0.395	Microbacterium	0.435	**0.005**
Gemmatimonadetes	1.775	0.153	Sphingobium	7.229	**0.000**
Fibrobacteres	0.713	0.643	Pseudonocardia	1.176	0.356
Candidatus_Cloacimonetes	4.395	**0.005**	Methylobacterium	2.119	0.094

Numbers in bold indicate passing the significance level test at P <0.05 or P <0.01.

At the genus level, overall dominant genera account for 34% to 36% of the relative abundance of all taxonomies, the dominant bacteria was *Streptomyces* (9.4%–11%), the secondary dominant bacterial genus were *Sphingomonas* (2.56%–5.08%), *Bradyrhizobium* (2.96%–4.09%), and *Pseudomonas* (2.59%–2.77%). Other genera occupy a smaller proportion (**Figure 2B**). One-way ANOVA showed that *Streptomyces, Sphingomonas, Bradyrhizobium, Nocardioides, Variovorax, Mesorhizobium, Mycobacterium, Microbacterium*, and *Sphingobium* showed significant differences among the seven precipitation gradients (**Table 2**). The distribution of *Streptomyces* from large to small was D2 (11.06%), D1 (10.72%), D3 (10.52), W3 (10.05%), CK (9.99%), W1 (9.79%), and W2 (9.4%), which decreased with the increase of precipitation. Further, *Sphingomonas* regularity of distribution from large to small was W2 (5.08%), W3 (4.69%), W1 (4.17%), CK (3.55%), D3 (3.26%), D1 (3.17%), and D2 (2.55%). The distribution pattern of *Bradyrhizobium* from large to small was W2 (4.09%), W3 (3.96%), CK (3.8%), W1 (3.78%), D2 (3.54%), D1 (3.23%), and D3 (2.96%), *Sphingomonas* and *Bradyrhizobium* both increased roughly with the increase in precipitation (**Figure 2B**).

#### Alpha diversity

**Figure 3:**
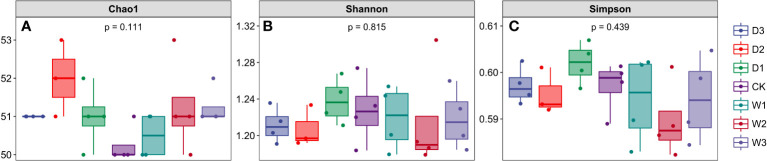
Alpha diversity index of soil bacterial community under seven precipitation gradients in the alpine steppe of Northern Tibet. **(A)** Chao1 index; **(B)** Shannon index; **(C)** Simpson index.

We calculated the Chao1 index, Shannon index, and Simpson index of soil microbial community under seven precipitation gradients, and the results showed that there were no significant differences among the three indexes under different water conditions, which also indicated the similarity of alpha diversity under seven precipitation gradients (**Figure 3**). The Chao1 index of all samples in the current study was more than 50, and the Shannon and Simpson indices of all samples were more than 1.16 and 0.59, respectively.This indicated that the number of species in the seven precipitation gradient communities was more than 50, but the community diversity was low (a larger Shannon index indicated a higher community diversity, while a larger Simpson index indicated a lower community diversity).

#### Beta diversity

**Figure 4:**
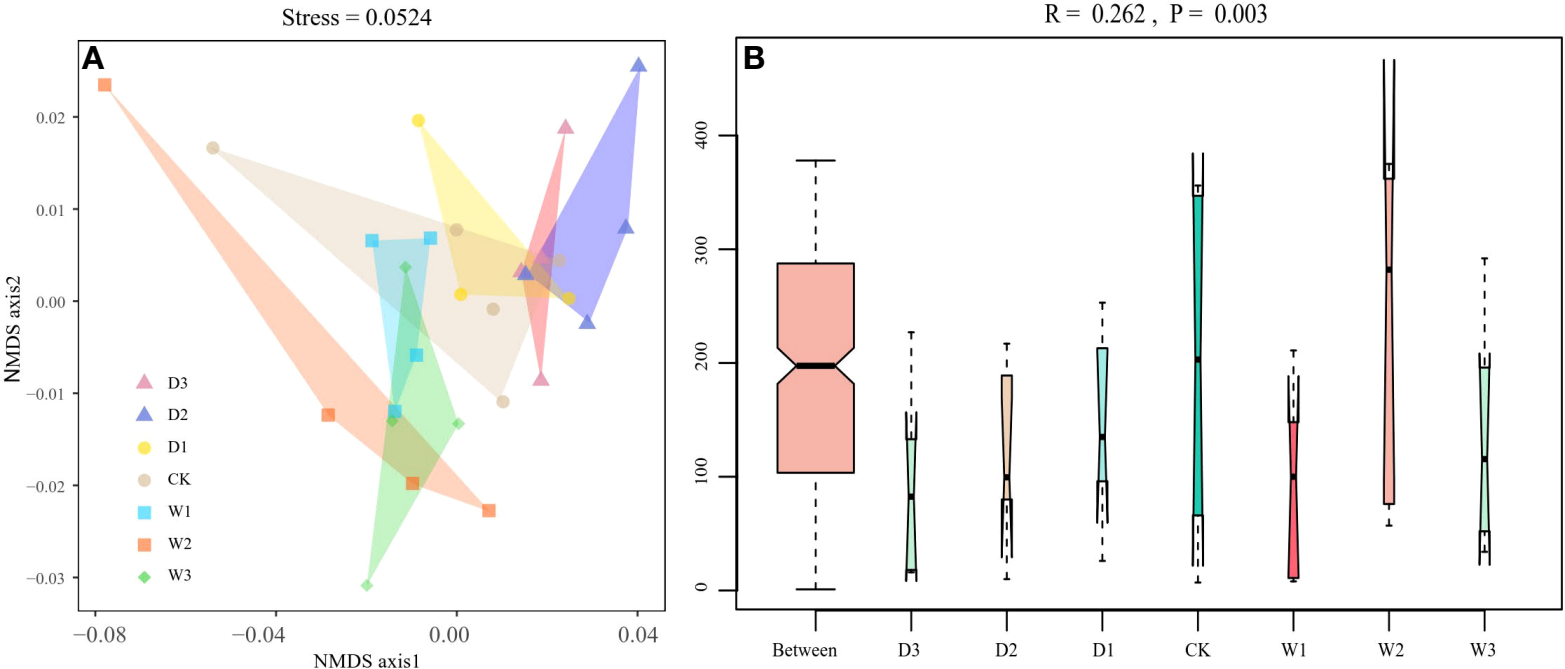
Beta diversity of soil bacterial community under seven precipitation gradients in the alpine steppe of Northern Tibet. **(A)** Non-metric multidimensional scaling (NMDS) analysis. Different shaped dots represent the samples from different alpine grassland types, and the bumps of the samples bound the faces of different colors. **(B)** Analysis of similarity (ANOSIM). Between reflects the differences between groups, and groups D3, D2, D1, CK, W1, W2 and W3 represent the differences within groups.

NMDS analysis was conducted based on Bray–Curtis dissimilarities to reflect the soil bacteria beta diversity of alpine steppe in the Northern Tibet. The results showed that the taxa of soil bacterial communities were significantly separated among seven precipitation gradients(Stress=0.0524, **Figure 4A**). For instance, the Bray–Curtis dissimilarity from the W2, W3 to the D2, D3 was relatively far, highlighting the significant difference of soil bacterial communities between them. In addition, the Bray–Curtis dissimilarity of W1, CK, and D1 was close, implying the similar soil bacteria community structures, especially the relative abundance of genus. Furthermore, the ANOSIM highlighted the differences in soil bacterial communities in alpine steppe (**Figure 4B** and **Table 3**). The soil bacteria community of D3 was substantially different from those of W1, W2, and W3 (*P*<0.05). Moreover, the soil bacterial community at D2 was different from that at W1, W3 (*P*<0.05). The results showed large differences in precipitation did change the structure of soil bacterial community, which was significantly different between the increase and decrease of 50% and 75% moisture.

**Table 3:** Analysis of similarities (ANOSIM) of soil bacteria in alpine steppe on the Northern Tibet.

Group	R	P-value	Sig
W1/D3	0.92	**0.024**	*
W1/CK	0.23	0.197	
W1/W3	-0.08	0.538	
W1/W2	0.09	0.186	
W1/D2	0.72	**0.041**	*
W1/D1	0.19	0.084	
D3/CK	0.05	0.295	
D3/W3	0.73	**0.024**	*
D3/W2	0.42	**0.037**	*
D3/D2	-0.03	0.558	
D3/D1	0.38	0.079	
CK/W3	0.20	0.212	
CK/W2	0.03	0.43	
CK/D2	0.06	0.296	
CK/D1	0.01	0.316	
W3/W2	-0.01	0.474	
W3/D2	0.63	**0.029**	*
W3/D1	0.18	0.106	
W2/D2	0.44	0.056	
W2/D1	0.38	0.064	
D2/D1	0.24	0.163	

Bold parts indicated significant differences at the p<0.05 level.

*Represented passed the test of significance of P<0.05.

### Co-occurrence network analysis of soil bacterial community

**Figure 5:**
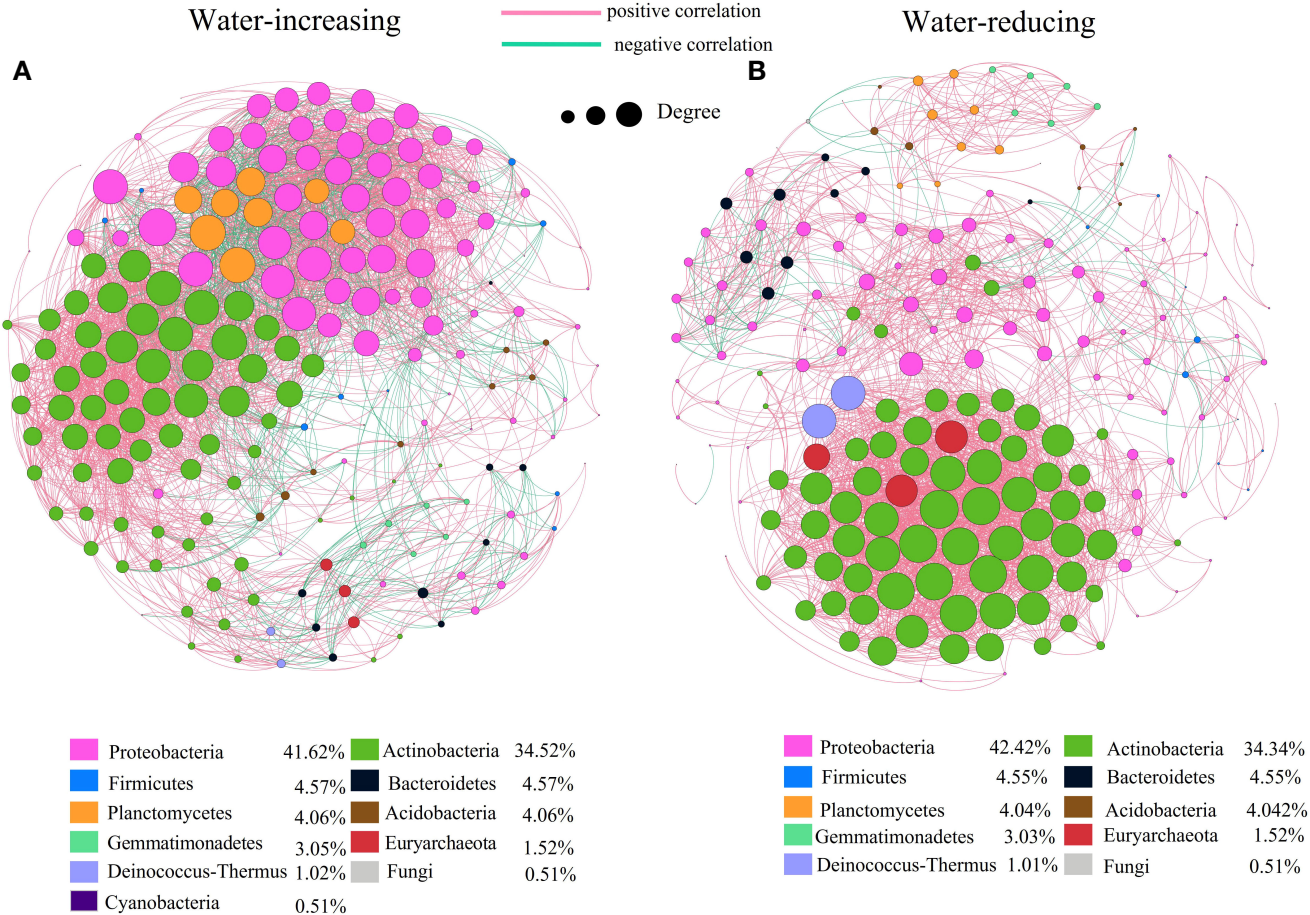
Network of co-occurring genera based on correlations analysis for water-increasing and water-reducing. A connection stands for a strong (Spearman’s p>0.7) and significant (p<0.01) correlation. The size of each node is proportional to the degree. Nodes colored by taxonomy, lines colored by positive and negative correlation, **(A)** water-increasing treatment, **(B)** water-reducing treatment.

In this study, network analysis was applied to illustrate the differences in soil bacterial community of water-increasing and water-reducing treatments in the growing seasons (**Figure 5**), and the network properties of soil bacterial communities under different water conditions were summarized in the **Table 4**. Water-increasing and water-reducing treatments had formed unique co-occurrence network of soil bacterial community, respectively. In the water-increasing treatment, the nodes and edges of the microbial network were 197 and 6654, respectively, with an average degree (AD) of 33.77 (**Figure 5A** and **Table 4**). In the water-reducing treatment, the microbial network had 198 nodes, but had fewer edges (4822) and a smaller average degree (24.354) (**Figure 5B**). This may indicate that the network structure of the water-increasing treatment was more closely connected, but the complexity of the network structure of the two water treatments was similar. In the co-occurrence network, the water-increasing treatment presented a shorter average path length (the shortest among all possible pairs of nodes) and a lower network diameter (the longest of the shortest paths among all pairs of nodes) (2.437 and 7.00) than those in water-reducing treatment (3.128 and 8.00), which reflected higher sensitivity and more rapid response to water addition. Furthermore, the higher clustering coefficient (0.708) of the water-reducing treatment was higher than water-increasing treatment (0.674). The modularity indices of two sample types were all greater than 0.40, which suggested that the co-occurrence networks of bacterial communities had a strong modular structure and complex species interaction across the two sample types in alpine steppe on the Northern Tibet.

**Table 4:** Network properties of soil bacterial communities for water-increasing and water-reducing treatments.

Treatment	Average degree (AD)	NetworkDiameter (ND)	Average path length (APL)	Clustering coefficient (CC)	Modularity (MD)	Nodes	Edges	Percentageofpositivecorrelation	Percentageofnegativecorrelation
Water-increasing	33.77	7	2.437	0.674	0.425	197	6654	80.49%	19.51%
Water-reducing	24.354	8	3.128	0.708	0.435	198	4822	96%	4%

In the network diagram of water-reducing treatment, positive correlation edges accounted for 96% (greater than water-increasing treatment, 80.49%) and negative correlation edges accounted for 4% (less than water-increasing treatment, 19.51%). The mutual exclusion effect of water-reducing treatment was small among species. The bacteria corresponding to the key species were predominantly *Proteobacteria* (41.62%,42.42%) and *Actinobacteria* (34.52%, 34.34%) in two types of treatment (**Figure 5**). However, the numbers of *Proteobacteria* in the network of water-reducing treatment was significantly reduced, indicating that the connectivity of *Proteobacteria* was gradually weakened (**Figure 5B**).

### Relationships between soil bacterial community and the environmental factors

**Figure 6:**
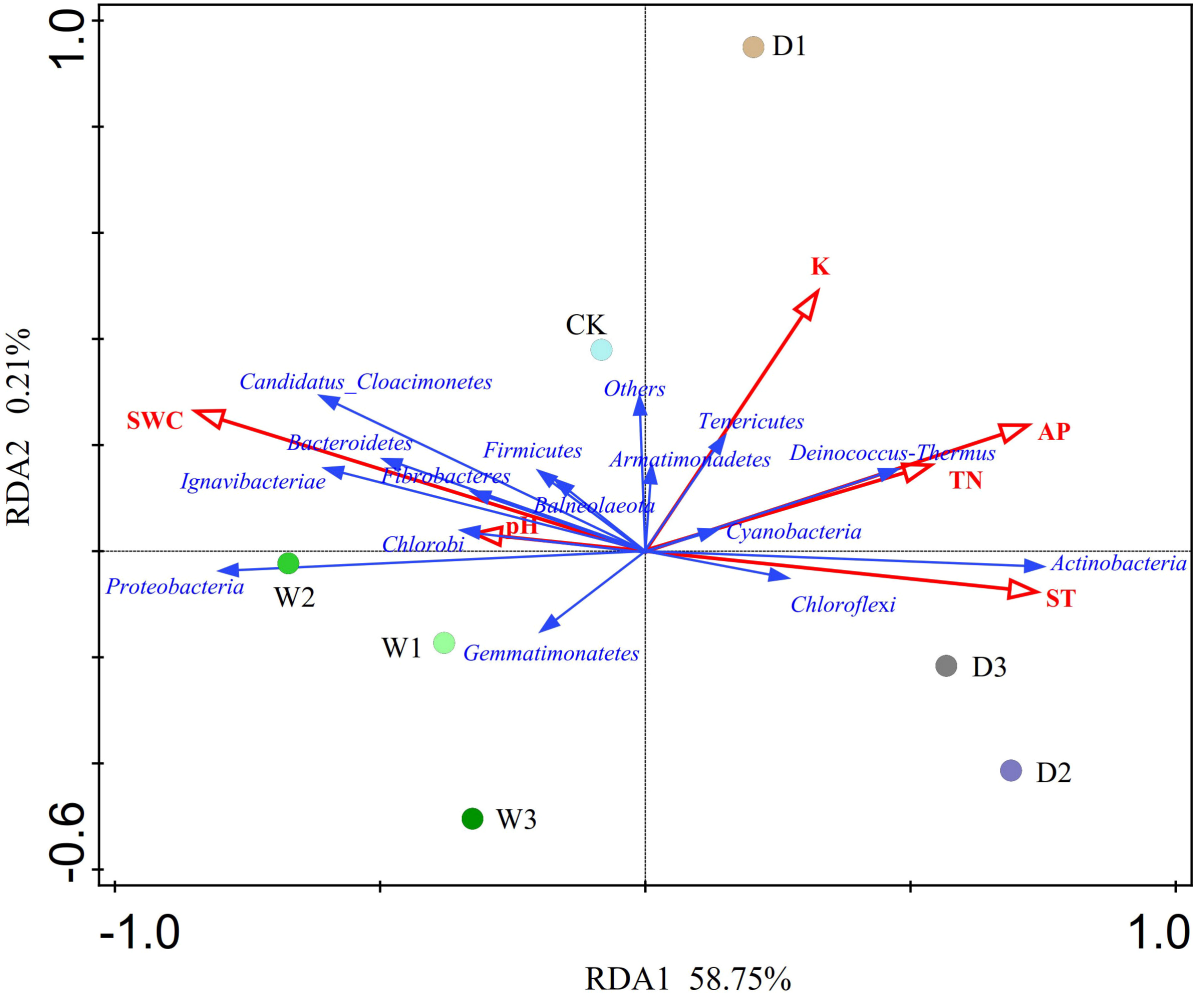
Redundancy analysis (RDA) of soil bacterial communities and environmental factors of different water conditions in alpine steppe on the Northern Tibet. Points denote sampling sites of seven precipitation gradients. The blue arrows represent soil bacteria (phylum), the red arrows represent environmental factors, and the cosine of the angle within two vector arrows represents the correlation between vectors. SWC, soil water content; AP, available phosphorus; K, kalium; ST, soil temperature; TN, total nitroge; P, phosphorus.

The RDA showed that the variations in the soil bacterial community were determined by environmental factors (**Figure 6**). The permutation test indicated significance in the ordination diagram (*P*=0.004). Environmental variables had an explanation rate of 58.96% for the soil bacterial community, with the first and second axes accounting for 58.75% and 0.21% of the variation, respectively. The results also revealed that correlations between each phylum and environmental variations. Environmental factors, especially SWC and AP were significantly related to the soil bacterial community (P < 0.05, **Table 5**). The difference in environmental factors in alpine steppes can explain the differences in soil bacterial communities among different water conditions. Further, the dominant factors for different soil bacteria were also different. For example, soil water content (SWC) was positively correlated to *Bacteroidetes, Candidatus_Cloacimonetes, Ignavibacteriae, Proteobacteria, Fibrobacteres, Chlorobi*, and *Firmicutes*, but negatively correlated to *Actinobacteria, Deinococcus-Thermus*, *Chloroflexi* and *Tenericutes*. Available phosphorus (AP) was positively correlated to *Deinococcus-Thermus, Actinobacteria*, and *Chloroflexi*, but negatively correlated to *Proteobacteria, Gemmatimonatetes* and *Chlorobi*. Total nitrogen (TN) was positively correlated to *Deinococcus-Thermus, Cyanobacteria* and *Actinobacteria*, but negatively correlated to *Gemmatimonatetes, Proteobacteria*, and *Chlorobi*. Soil temperature (ST) was positively correlated to *Actinobacteria* and *Chloroflexi*, but negatively correlated to *Proteobacteria, Candidatus_Cloacimonetes, Bacteroidetes*, and *Ignavibacteriae*. In addition, the soil bacterial abundance at different points also showed variability of water conditions. For instance, the projection point of the corresponding sample points in W2 on the corresponding vector of *Proteobacteria* was ahead of other points, suggesting that *Proteobacteria* has greater abundance potential in the W2 than in other points.

**Table 5:** Significance of explanatory variables and the variance values in redundancy analysis (RDA).

Environmental factor	Explanation (%)	Contribution (%)	Pseudo-F	p
SWC	42.2	71.5	19.0	**0.002**
AP	8.8	15.0	4.5	**0.034**
pH	4.5	7.6	2.4	0.128
K	3.3	5.6	1.8	0.174
ST	0.1	0.2	<0.1	0.886
TN	<0.1	0.1	<0.1	0.934

Bold parts indicated significant differences at the p<0.05 level.

## Discussion

### Responses of soil microbial community to precipitation change

Soil microbial communities have different response strategies to different precipitation gradients in semi-arid ecosystems (Placella et al., 2012). This is because precipitation directly affects soil water availability and indirectly affects plant productivity, carbon allocation and above-ground and below-ground litter input, which ultimately changes soil microbial communities, especially in semi-arid regions with water shortage (Sierra et al., 2017; Sihi et al., 2018; Zhou et al., 2018). In this study, we found that *Proteobacteria* and *Actinobacteria*, which accounted for 45–52% and 37–45% of the seven precipitation gradients at the phylum level, respectively, were the dominant bacteria, while Firmicutes were the secondary dominant bacteria, and their relative abundance was only about 2% (**Figure 2A**). Actinobacteria constitute one of the largest bacterial phyla in many types of soil ecosystems (Barka et al., 2016). They often live as plant commensals and nitrogen-fixing symbionts in soil (Fitzpatrick et al., 2018), participating in the formation of soil organic matter and biogeochemical cycles (Yu et al., 2020). *Proteobacteria* were one of the major phyla of ammoniated microorganisms and are also abundant in grassland ecosystems (Chen et al., 2020). *Proteobacteria* and *Actinobacteria* are the dominant bacteria in grassland, which is consistent with previous studies (Zeng et al., 2017; Nan et al., 2020). However, their relative abundance ratio of *Proteobacteria* and *Actinobacteria* were different due to environmental differences caused by different study areas and ecosystem types. For example, a study of the high-altitude Gangotri soil ecosystem confirmed that *Proteobacteria* and *Actinobacteria* accounted for 38.49% and 14.48%, respectively (Kumar et al., 2019). *Proteobacteria* and *Actinobacteria* accounted for 47.19% and 42.2% of the soil microbial communities in the alpine grassland of northern Tibet, respectively (Fu et al., 2022). Ding et al. (2013) found that *Actinomycetes* were more dominant in arid and semi-arid habitats than in agricultural ones (Ding et al., 2013).

The relative abundance of *Proteobacteria* in water-increasing treatment was significantly higher than that in water-reducing treatment, while *Actinobacteria* was significantly lower than that in water-reducing treatment (**Figure 2A** and **Table 1**). This may be due to the different evolutionary and physiological adaptation mechanisms that made the phylum of soil bacteria respond differently to precipitation changes (Cregger et al., 2012). *Proteobacteria* belong to a group of water sensitive bacteria and keep strong consistent change with precipitation. These groups are generally copiotrophic and fast-growing bacteria, and respond to unstable carbon availability due to changes in precipitation (Fierer et al., 2009). *Actinomycetes* are Gram-positive bacteria, which have a strong tolerance to drought and can grow in dry conditions. Therefore, the relatively high abundance of *Actinomycetes* in the water-reducing treatment may be related to their stronger cell walls, ability to produce spores and grow in filamentous form, which helps to effectively reduce the damage caused by drought and high temperature (Koyama et al., 2018; Li et al., 2018).

At the genus level, *Streptomyces* was confirmed to be the dominant genus in seven precipitation gradients (9.4%–11%), but it decreased with increasing precipitation. The proportion of *Sphingomonas* and *Bradyrhizobium* was very small (2.56%–5.08% and 2.96%–4.09%, respectively), but both increased along with the increase in precipitation (**Figure 2B**). *Streptomyces* is the largest bacterial group and is widely distributed in nature, especially in soil (Barka et al., 2016). *Streptomyces* was previously reported to be the dominant bacterium in the alpine grassland environment of northern Tibet (Yin et al., 2016), which is consistent our data. However, *Streptomyces* did not always increase with the amount of water available, which may be driven by competitive interactions between soil fungi and soil bacteria or predator-prey dynamics between soil microbes and soil protozoa (Cregger et al., 2012). *Sphingomonas* was the main antibacterial agent in the soil community, which had an inhibitory effect on pathogenic plant fungi (Kyselková et al., 2009; Wachowska et al., 2013). *Bradyrhizobium* is a common soil microorganism that establishes a symbiotic relationship with plant roots and fix nitrogen (Chen, 2016). *Sphingomonas* and *Bradyrhizobium* were higher in water-increasing treatment, indicating that they also maintained a higher consistency with soil moisture.

The α-diversity mainly measures the diversity of microbiota within individuals, while β-diversity is an index to characterize the similarity of microbial composition between individuals (Yang et al., 2022).We analyzed soil microbial community diversity under seven precipitation gradients and found that there were no significant differences in the three α-diversity indices (**Figure 3**), indicating that the α-diversity of soil bacterial communities was not affected by short-term precipitation changes. Bachar et al. (2010) and Wang et al. (2014c) showed that changes in precipitation did not alter bacterial community diversity in Mediterranean, semiarid, and arid regions, and they found that bacterial richness fluctuated little between seasons with different water availability, which was consistent with our results (Bachar et al., 2010; Wang et al., 2014c). Zhang et al. (2019b) found that the frequency of *Acidobacteria* decreased with the addition of water (Zhang et al., 2018a), which may be because the number and abundance of bacterial communities in arid and semi-arid areas were low, and the composition of bacterial communities was relatively similar (Wu et al., 2020). Compared to other soils, Yang and Li, (2020) pointed out that grassland soils had a greater ability of microorganisms to maintain their functions in response to repeated water stress (Yang and Li, 2020).In addition, some studies have shown that changes in precipitation have no significant effect on bacterial community composition in surface soil, but does affect bacterial abundance, which is consistent with our results (Wang et al., 2014c; Shi et al., 2018).The variation of precipitation had no significant effect on the composition of soil bacterial community at phylum level, but only affected a few soil water-sensitive species (Jia et al., 2021). Bray–Curtis dissimilarities represent β-diversity, and the results revealed the differentiation of soil microbial communities among the seven precipitation gradients of alpine grasslands. The significant difference in soil bacterial communities between D2, D3, and W2, W3 was demonstrated because the Bray–Curtis distance from D2, D3 to W2, W3 was relatively far (**Figure 4**). These results indicated that β-diversity had a significant effect on the treatments with large precipitation variations ( ± 50% and ±75%). This indicated that the composition of microbial communities among them was quite inconsistent. Sudden changes in soil moisture stress microorganisms because they must expend energy to regulate the osmotic pressure of their microenvironment (Evans and Wallenstein, 2012) and affect REDOX regulation (Bubier and Moore, 1994). In addition, climatic factors can alter soil geochemistry and thus affect microbial community structure. In general, the inconsistent responses of microorganisms to precipitation changes may be caused by ecosystem specific responses, historical precipitation mechanisms, or indirect water-induced environmental factors (She et al., 2018).

### Differences of co-occurrence network between water-increasing and water-reducing treatment

Soil microorganisms exist in complex ecological networks, and also show different types of network relationships, including symbiosis, competition, predation, partially beneficial, or partially harmful relationships (Faust and Raes, 2012). Understanding their co-occurrence patterns can help to understand microbial interactions from a new perspective (Ma et al., 2016a), and explain microbial spatial niches (Freilich et al., 2018), which is of great significance for understanding biodiversity and community stability (Zhang et al., 2019b). Our results showed that there was little difference in the nodes between the two soil sample types, while the edges in the water-reducing treatment was much smaller than that in the water-increasing treatment (**Figure 5** and **Table 4**). This indicated that the stability (complexity) of bacterial communities in two soil sample types was similar, but the communities in the water-increasing treatment were more closely connected and the connections in the water-reducing treatment were more distant (Hu et al., 2022). This suggested that the interaction, or at least the potential of mutual influence, between species in water-increasing treatment may be more intense or effective, indicating that synergies or competition between different bacterial species may be enhanced by up-regulating wetting disturbance in this study (He et al., 2017). In addition, the average path length and network diameter of water-increasing treatment were lower, which may indicate higher sensitivity and faster response of water addition among species under wet conditions (Jia et al., 2021). The longer average path distance indicated a lower sensitivity and slower response to water reduction (**Table 4**). This might be related to the adaptation of bacterial communities to the drought conditions of alpine steppe (Jia et al., 2021). Both water-increasing and water-reducing treatments had stable modular structures, which confirmed that short-term drought or wetting disturbance did not damage the stability of microbial community in the region. The weak interaction was stronger with increasing water, which indicated that the ecological network among species treated with increasing water was more stable (Guan et al., 2021). A precipitation gradient experiment in Inner Mongolia desert steppe also confirmed that the robustness of microbial networks increased with increasing precipitation (Li et al., 2022a). However, we found that decreased precipitation enhanced positive interactions between microbial species (**Figure 5** and **Table 4**), possibly because limited soil water availability may have improved interactions and cooperation between functional groups of soil biota (Holtkamp et al., 2011); Another explanation is that the ecological niches of microbial groups may overlap under the soil moisture condition of water shortage and reduced precipitation (Hernandez et al., 2021).The degree of *Proteobacteria* decreased in water reduction treatment, and the role of module center or connector was weakened, which may be due to characteristics of *Proteobacteria.* Importantly, *Proteobacteria* belong to a group of water sensitive bacteria, which maintain a strong and consistent change with precipitation. These groups are generally copiotrophic, fast-growing bacteria, and respond to unstable carbon availability due to changes in precipitation (Fierer et al., 2009; Li et al., 2022a). In all network interactions, most of the core nodes were corresponding to *Proteobacteria* and *Actinomycetes*, and their relative abundance in soil microbial pathways was also the highest among all detected phyla (Fu et al., 2022). Therefore, *Proteobacteria* and *Actinomycetes* are the most important key bacterial species in the alpine grassland of northern Tibet, which are crucial for maintaining the stability of microbial communities in the study area.

### The key factor driving the change of soil microbial community

Changes in precipitation lead to changes in soil water content (Zhang et al., 2019a), which leads to changes in other soil physical and chemical indexes. Subsequently, this leads to a change in soil redox conditions, which affects the structure and diversity of soil microbial community (Vanegas et al., 2013). Because changes in precipitation affect the soil available water and material migration, this results in changes the substrate supply of soil microorganisms (Apostel et al., 2015; Ren et al., 2018a). We found that soil temperature promote growth of *Actinomycetes* (Yang et al., 2022) and that soil moisture was negatively correlated with *Actinomycetes*. Because *Actinomycetes* are aerobic bacteria, soil moisture content was reduced in water-reducing treatment, evaporation was increased, and there was more oxygen content in the soil air, all of which are conditions that are conducive to *Actinomycetes* growth (Li et al., 2020). Other studies have shown that *Actinomycetes* can adapt to any extreme environment (such as drought, infertility and malnutrition), mainly determined by their diversity (Grover et al., 2016; Takahashi and Nakashima, 2018; Das et al., 2021), which is consistent with our results (Mickan et al., 2018). Another study of three genomes with similar 16S rRNA sequences of *Acidobacteria* revealed that the presence of fiber synthesis genes and a large number of novel high molecular excretion proteins proved that *Acidobacteria* had certain drought tolerance (Ward et al., 2009). Nitrogen elements is widely distributed limiting environmental factors in terrestrial ecosystems, and the dynamic change of soil nitrogen content has an important impact on the ecosystem (Wang et al., 2014b). In the current study, we found that total nitrogen was positively correlated to *Deinococcus-Thermus*, *Cyanobacteria* and *Actinobacteria*, which was consistent with a previous study on the newly reclaimed mudflat paddy soils (Li et al., 2022b). The reason was that nitrogen could promote the increase of the relative abundance of some bacterial communities and induced a shift in bacterial community composition towards a more copiotrophic type (Wu et al., 2021; Yao et al., 2021; You et al., 2021). In addition, this study found that there was a certain degree of correlation between available phosphorus (AP) and *Actinomycetes, Deinococcus-Thermus, Chloroflexi, Proteobacteria, Gemmatimonatetes* and *Chlorobi*. AP was an important nutrient in the soil of the alpine steppe in Northern Tibet, and its distribution in the soil was affected by precipitation change, which also had some effects on microbial species.

Changes in soil temperature might directly or indirectly kill microorganisms and reduce microbial abundance by limiting soil water and nutrient availability; But the tolerant/adaptive species that survived increased their advantage (Walker et al., 2006; Wilson and Walker, 2010). Secondly, the amount of substrate at higher soil temperatures may be very conducive to the rapid proliferation of microorganisms (Sharma et al., 2006). Zhang et al. (2017) pointed out that the high altitude area was only suitable for the survival of cold-tolerant bacteria, which reflected the restrictive effect of temperature on microbial flora (Zhang et al., 2017). In the current study, soil temperature was significantly different under seven water gradients due to the precipitation control experiment (supplement), which also affected the abundance of bacterial communities. At present, different research groups have drawn different conclusions on the influence of soil environmental factors on microbial community (Sun et al., 2012; Liu et al., 2016). Therefore, the response of soil microbial community to precipitation change in the alpine steppe of northern Tibet needs to be further studied.

## Conclusion

In general, we can draw the following four conclusions from current studies. (1) *Actinomycetes* and *Actinomycetes* are the most important key bacterial taxa in the alpine steppe of northern Tibet and they play a crucial role in maintaining the stability of microbial community. (2) The α-diversity of soil microbial community is not significantly affected by precipitation change, while the β-diversity is significantly affected by drastic changes in water supply. (3) In the co-occurrence network, the communities in the water- increasing treatment were more closely connected than those in the water-reducing treatment. The network patterns of water-increasing/reducing treatment both showed strong positive correlation, but the water-reducing treatment was greater. (4) SWC and AP were key environmental factors affecting soil microbial community composition. Furthermore, the results also highlighted the tolerance of *Actinobacteria* to arid environments and the positively sensitive response of *Proteobacteria* to soil moisture. The results of this study are key to aiding our understanding of the response of the soil microbial community structure to precipitation changes in northern Tibet. Moreover, these data provide a more in-depth reference for studying the driving mechanism of global climate change on soil microbial community and grassland ecosystem in alpine grassland.

## Data availability statement

The original contributions presented in the study are publicly available. This data can be found here: NCBI, PRJNA881773.

## Author contributions

YY designed the experiment. XQL, LF, and YL carried out the experiment and collected the soil samples. XQL analyzed the data, and completed the writing and modification of the first draft. XYL and YY participated in the discussion of the content of the first draft and put forward valuable suggestions. All authors contributed to manuscript revision, read, and approved the final submitted version of the manuscript.

## Funding

This research was supported by the National Natural Science Foundation of China (41871049 and 41877338).

## Acknowledgments

We would like to thank the Xainza Alpine Grassland and the Wetland Ecosystem Observation and Experimental Station for providing us with the experimental site and the station staff, as well as Yiduo for his help in the sampling work.

## Conflict of interest

The authors declare that the research was conducted in the absence of any commercial or financial relationships that could be construed as a potential conflict of interest.

## Publisher’s note

All claims expressed in this article are solely those of the authors and do not necessarily represent those of their affiliated organizations, or those of the publisher, the editors and the reviewers. Any product that may be evaluated in this article, or claim that may be made by its manufacturer, is not guaranteed or endorsed by the publisher.
